# Elevated Temperatures Disrupt *Wolbachia*‐Induced Feminisation and Reshape Microbial Community Dynamics Across Generations in a Spider Host

**DOI:** 10.1111/mec.70371

**Published:** 2026-05-08

**Authors:** Virginija Mackevicius‐Dubickaja, Jennifer A. White, Ellen E. Williams, Eyal Klement, Yuval Gottlieb, Matthew R. Doremus

**Affiliations:** ^1^ Koret School of Veterinary Medicine, The Robert H. Smith Faculty of Agriculture, Food and Environment The Hebrew University Rehovot Israel; ^2^ Department of Entomology University of Kentucky Lexington Kentucky USA; ^3^ Department of Entomology University of Illinois at Urbana‐Champaign Urbana Illinois USA

**Keywords:** bacterial titre, co‐infection, maternal transmission, microbial interactions, reproductive parasites, temperature

## Abstract

Longitudinal microbial interactions within a host are challenging to study, leading to a focus on constructed microbial communities in vitro settings. Here, we take advantage of a naturally defined microbial community within a spider host to study how elevated temperatures influence microbial dynamics and phenotypes across host generations. The spider *Mermessus fradeorum* hosts up to five endosymbionts, including a *Wolbachia* strain, W1, which induces feminisation, causing genetic males to develop as phenotypic females, skewing sex ratios and promoting symbiont spread. Despite this, *Wolbachia 1* persists at intermediate frequencies in wild populations. We hypothesised that elevated temperatures might reduce penetration of the feminisation phenotype, potentially by altering symbiont dynamics and maternal transmission. We exposed spiderlings co‐infected with *Wolbachia 1* to elevated temperatures for one generation and measured feminisation rate, symbiont transmission, and titre across three generations. Feminisation was unaffected in the exposed (F1) generation but declined in subsequent generations (F2, F3) that were not directly exposed. This multigenerational effect was linked to shifts in symbiont community dynamics: low feminisation coincided with high abundance of one symbiont, *Rickettsiella*, a decline in *Wolbachia 1* transmission, and complete loss of another symbiont, *Tisiphia*. Our findings demonstrate how environmental history shapes the evolutionary stability of microbial communities and their induced phenotype in their natural host.

## 1 Introduction

Arthropods host a diverse array of maternally transmitted reproductive parasites that manipulate host reproduction to favour the production of infected females (Katsuma et al. 2022; Hurst and Frost 2015; Adachi‐Hagimori et al. 2008). These symbionts are often found within a community of other maternally transmitted symbionts that may affect their induced phenotypes (Heyworth et al. 2020; Li et al. 2020; McLean et al. 2018). While studying microbial interactions within animal hosts is usually restricted to reduced in vitro systems (van Leeuwen et al. 2023), arthropod‐endosymbiont communities enable in vivo testing of interactions and effects under environmental constraints (e.g., Proctor et al. 2024).

Environmental factors, particularly temperature, can modulate endosymbiont titre (e.g., Gharabigloozare and Bleidorn 2022) and the efficiency of maternal transmission (e.g., Hague et al. 2022), thus affecting symbiont persistence in the host (Hague, Mavengere, et al. 2020) and the expression of reproductive parasitism phenotypes (Martins et al. 2023). Often, the expression of these phenotypes is stronger at lower temperatures (Nasehi et al. 2022; Ning et al. 2019; Anbutsu et al. 2008; Hurst et al. 2001), although there are several studies demonstrating a positive correlation between increased temperature and phenotype expression (Zhou et al. 2019; Montenegro and Klaczko 2004). Thus, thermal conditions may either enhance or suppress symbiotic reproductive manipulations (Proctor et al. 2024; Hu et al. 2023; Tougeron and Iltis 2022), consequently influencing host physiology and behaviour (Hague, Caldwell, and Cooper 2020; Truitt et al. 2018; Feldhaar 2011).

*Wolbachia*, the most studied reproductive manipulator, induces several thermally labile host phenotypes to enhance its transmission to female offspring, including the feminisation of genetic males, which skews the sex ratio almost exclusively toward females (Kaur et al. 2021). Higher temperatures can reduce *Wolbachia's* maternal transmission efficiency and the strength of reproductive parasitism (Hu et al. 2023; Ning et al. 2019; Zhou et al. 2019). In some cases, like the moth *Ostrinia scapulalis*, temperature‐induced feminisation failure can result in increased numbers of male progeny (Sakamoto et al. 2008). In others, including the isopod *Armadillidium vulgare* and the butterfly *Eurema hecabe*, feminisation failure can be detrimental to the host by producing sterile intersex individuals or causing host mortality (Narita et al. 2007; Rigaud and Juchault 1998). Additionally, *Wolbachia* can also be part of multi‐symbiont communities that may affect its reproduction phenotype via specific symbiont interactions (Engelstädter et al. 2004; White et al. 2011; Zhu et al. 2012). However, the long‐term, multigenerational consequences of heat exposure for the stability of *Wolbachia*‐induced reproductive manipulation under changing environmental conditions in a community context remain largely unknown.

The dwarf spider *Mermessus fradeorum* (Araneae: Linyphiidae) hosts up to five heritable symbionts, including a *Rickettsiella* that causes cytoplasmic incompatibility (CI), a reproductive manipulation that causes embryo death when infected males mate with uninfected females, *Tisiphia* (formerly *Rickettsia*; Davison et al. 2022), and three strains of *Wolbachia* (W1‐3) (Curry et al. 2015; Rosenwald 2020; Rosenwald et al. 2020). Spiders whose infection includes *Wolbachia* strain 1 (W1) are feminised and produce almost exclusively female offspring (Mackevicius‐Dubickaja et al. 2025). The presence of *Wolbachia 1* is essential for feminisation, but feminisation rates vary with different symbiont combinations. While the phenotypic effects of *Wolbachia* strains 2 (W2) and 3 (W3) remain unknown, their presence appears to enhance feminisation, as the highest and most consistent feminisation rates are observed in communities containing all five symbionts (Mackevicius‐Dubickaja et al. 2025). Yet the quintuple assembly is present only at intermediate frequencies (19%) in field‐collected spiders, and other *Wolbachia 1* infection combinations occur even less frequently (Rosenwald 2020). Unlike *Wolbachia 1*, CI‐inducing *Rickettsiella* remains nearly fixed in the population (Rosenwald 2020) despite exhibiting a temperature‐sensitive CI phenotype (Proctor et al. 2024). The presence of *Wolbachia 1* at intermediate frequencies in field‐collected spiders suggests it, too, might experience temperature sensitivity, which might constrain its spread.

Given the known temperature sensitivity of *Wolbachia* in other systems and its residence within a symbiont community, we hypothesise that exposure to elevated temperatures in a single host generation might affect microbial community dynamics, which in turn would influence *Wolbachia 1* feminisation and symbiont transmission rates, titre, and relative abundance across multiple host generations. For this, we tested how exposure to elevated temperatures during a single spider generation affected numbers of female offspring, symbiont prevalence, titre, and relative abundance across three generations. We found that exposure to elevated temperatures did not immediately affect feminisation of treated spiders but disrupted *Wolbachia 1*‐induced feminisation in later generations, even though those later generations did not directly experience warm temperatures. These multi‐generational changes seem to be driven by shifts in symbiont community composition: *Wolbachia 1* abundance increased immediately following exposure to warm temperatures and was unexpectedly associated with reduced feminisation in offspring. In unfeminised offspring, *Wolbachia 1* abundance subsequently declined, coinciding with an increase in *Rickettsiella* abundance. This shift links the elevated relative abundance of *Rickettsiella* with a reduced likelihood of feminised brood within the same generation. Additionally, exposure to warm temperatures further changed the composition of the symbiont community by reducing *Wolbachia 1* transmission rates and causing the complete loss of *Tisiphia*. Altogether, temporary exposure to elevated temperatures reduces the feminising capability of *Wolbachia 1* for multiple host generations and destabilises the expanded symbiont community in *M. fradeorum* . These temperature effects may restrict the efficacy of *Wolbachia* feminisation and the spread of this symbiont in the *M. fradeorum* population.

## 2 Materials and Methods

### 2.1 Origin and Maintenance of *Mermessus fradeorum* Spiders

*Mermessus fradeorum* spider cultures used in this study were initially collected from alfalfa ( *Medicago sativa* ) in Kentucky, USA. Spiders were maintained at 20°C in 4 cm diameter plastic cups with moistened plaster at the bottom for humidity (Rosenwald et al. 2020). Immature spiders were fed collembola ( *Sinella curviseta* ) until they were large enough to consume one wingless *Drosophila melanogaster* twice a week.

*Mermessus fradeorum* spiders collected from North American field populations naturally harbour up to five heritable symbionts: *Rickettsiella* (R), *Tisiphia* (T), and three *Wolbachia* strains (W1, W2, W3). For simplicity, infection compositions are denoted by concatenated symbiont abbreviations. Lines used in the study were either “feminised” (infected with *Wolbachia 1* and one to four more symbionts) or uninfected. Feminised spiders were routinely screened for confirmation of symbiont presence using diagnostic PCR (Mackevicius‐Dubickaja et al. 2025). Uninfected spider lines lacking heritable symbionts had been generated previously by treating spiders with a fine spray of tetracycline (0.1%) and ampicillin (0.1%) until sub‐adulthood (Rosenwald et al. 2020). All lines produced via antibiotic treatments were maintained in the lab for at least 5 generations prior to use in experiments and confirmed to be symbiont‐free using diagnostic PCR.

### 2.2 Experimental Design

**FIGURE 1:**
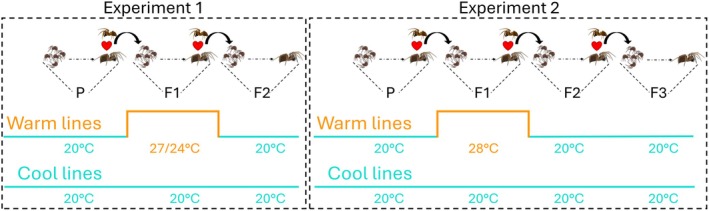
Experimental designs used to test the multi‐generational effects of elevated temperatures on *Wolbachia*‐induced feminisation in *Mermessus fradeorum* spiders. In both experiments, females were mated to uninfected males. Cool‐lines (teal) were continuously reared at 20°C, and Warm‐lines (orange) were exposed to a higher rearing temperature in the F1 generation. Elevated temperatures included an oscillating cycle of 27/24°C (day/night) in the first experiment (left) and a constant 28°C in the second experiment (right). All other generations were reared at 20°C. For estimation of symbiont transmission and titre, mated females (Exp. 1: P and F1; Exp. 2: P, F1, and F2) were preserved in 95% ethanol following egg mass deposition, while F2 and F3 offspring, from Exp. 1 and 2, respectively, were preserved in 95% ethanol following their adult moult. Letters below the spider icons represent generations per experiment. Male and female spider illustrations are courtesy of J. A. White. Spiderling images were adapted from Easy Drawing Guides and modified in Microsoft PowerPoint.

To test the effect of temperature exposure on *M. fradeorum* feminisation, we performed two multi‐generational experiments in which *Wolbachia 1* co‐infected (feminised), and uninfected (not feminised) spiders were exposed to a warm (Exp1: 27/24°C; Exp2: 28°C) or cool (20°C) temperature treatment during their development (Figure 1). Experiment 1 was conducted as an initial test of temperature effects, based on prior evidence suggesting that bacterially induced feminisation responds rapidly to thermal stress (Herran et al. 2020; Badawi et al. 2015; Negri et al. 2009; Narita et al. 2007). Accordingly, this experiment was carried through the F2 generation to assess both immediate (F1) and short‐term transgenerational (F2) responses. The warm treatment in Experiment 1 consisted of an oscillating 27/24°C day/night cycle (16 h day: 8 h night), reflecting the average high/low surface temperatures spiders experience during a long‐day summer cycle in Kentucky, USA.

Based on results from Experiment 1, which showed that feminisation was not affected in the exposed generation (F1) but was reduced in the subsequent generation (F2), we conducted Experiment 2 as a follow‐up under slightly modified conditions. In this second experiment, spiders were exposed to a constant 28°C, representing daytime surface temperatures recorded in Kentucky, USA. The design was extended to the F3 generation to assess whether feminisation recovers after the observed decline. In both experiments, cool‐treated spiders remained at 20°C for their entire development. Because both experiments produced consistent phenotypic patterns, data were combined for phenotypic analyses to increase statistical power. The uninfected spiders are a control for temperature effects on spider reproduction.

In both experiments, we generated egg masses from crosses between male and female spiders kept at 20°C (P generation; *n* = 22–27 egg masses per experiment and infection group). Upon hatching at 20°C, we then moved a subset of the F1 generation (*n* = 10–14 broods per experiment and infection group) to a warmer temperature (warm lines), while the remaining F1 spiderling broods (*n* = 11–13 broods per experiment and infection group) were kept at 20°C (cool lines). The number of spiderlings retained per brood varied between *n* = 4–26 (Experiment 1) and *n* = 4–8 (Experiment 2). Egg masses producing fewer than four offspring were removed from the experiment. Warm F1 spiders remained at the elevated temperature until one week following their adult moult. To determine if warm temperatures caused an immediate effect on the feminisation phenotype, F1 spider sex was identified by confirming the presence of enlarged pedipalps (males) or the epigynum (females) on adult spiders. To determine if temperature had multi‐generational effects on offspring feminisation, we selected a subset of F1 females from both temperature lines (warm and cool) to mate with uninfected males. We kept all females at 20°C post‐mating for deposition of F2 egg masses (*n* = 10–15 females for all Exp1 and Exp2 F1 crosses). All F2 offspring developed at 20°C, regardless of F1 temperature treatment. We then recorded the F2 offspring sex ratio at adulthood by identifying the sex of up to eight randomly selected offspring per mother. In the second experiment, we followed experimental spider lines for an additional generation by crossing a subset of the F2 female spiders with uninfected males (*n* = 18–22 females for all Exp2 F2 crosses). All F3 spiders continued to develop at 20°C until they reached adulthood, at which point their sex ratio was determined as in the prior generation. This experimental design allowed us to test not only the immediate effect of developmental warm temperature exposure on spider feminisation, but also the effect of that same exposure on the feminisation of subsequent generations.

For both experiments, mated females were preserved in 95% ethanol following egg mass deposition, while offspring used to calculate sex ratio were preserved in 95% ethanol following their adult moult. Preserved spiders were kept at −20°C for gDNA extractions using the DNeasy Blood and Tissue Kit (Qiagen) according to the manufacturer's protocol. Extracted DNA was used to estimate symbiont transmission rate using diagnostic PCR and titre using digital PCR (dPCR).

Raw data of all the experimental procedures (proportion female ratio, transmission rate, and digital PCR) are available in Data S1.

### 2.3 Diagnostic and Digital PCR, and Symbiont Titre Measurement

We used diagnostic PCR to determine symbiont infection status of *M. fradeorum* samples from all generations of Experiment 1 (P, F1, and F2) and for a subset of samples from Experiment 2. Digital PCR (dPCR) was used to quantify the symbiont genome across generations (P, F1, F2, and F3), using a subset of individuals from Experiment 2. Primer and probe sequences used in this study are provided in Table S1. Experiment 1 was originally designed to assess infection status using standard PCR, and samples were not preserved for downstream quantification of symbiont titre or relative abundance. Following the observation that feminisation was reduced in the F2 generation, Experiment 2 was conducted with the additional aim of linking phenotypic outcomes to symbiont abundance and community structure, and samples were therefore collected and preserved accordingly for molecular analyses.

To normalise symbiont load relative to host cell count, we also quantified the host *18S rRNA* housekeeping gene in each sample. Assays were performed using short‐specific primers and probes, following the same protocol described in Mackevicius‐Dubickaja et al. (2025). Both symbiont and host gene targets were quantified using the Qiagen QIAcuity Probe PCR Kit on a QIAcuity One‐plate digital PCR system (QIAcuity One, 5plex instrument, Qiagen, Germany).

### 2.4 Sex Ratio Statistical Analyses

Data from the two independent experiments (Experiment 1 and Experiment 2) were first analysed for consistency between experiments prior to their inclusion in a combined analysis. We fitted a generalised linear model (GLM) with a quasibinomial distribution to account for overdispersion and compared the proportion of female offspring across spider broods using a fully factorial model that included experiment, F1 temperature, generation, and infection as factors. F3 spiders were not included in this initial analysis, as this generation was unique for Experiment 2. Following confirmation that the experiment was not a significant factor in the initial model (ΔDeviance = 1.624, df = 1, *p* = 0.307), we pooled the data from both experiments to increase statistical power and improve the precision of parameter estimates. We then reran the fully factorial model with F1 temperature, generation, and infection as factors. To gain further clarity about the nature of higher‐order interactions in the full model, we ran separate GLM models for each generation (F1, F2, F3), with F1 rearing temperature, infection group, and their interaction as predictors. Diagnostic screens of F2‐infected mothers using PCR and dPCR revealed that several experimental lines had lost *Wolbachia 1* following heat treatment; to account for this loss of *Wolbachia 1*, we performed a second GLM analysis of F3 sex ratios in which we removed spider lines that had lost *Wolbachia 1*. Sex ratio analyses were conducted in R v4.4.0 (R Core Team 2024).

### 2.5 Transmission Rate

To test how temperature affected symbiont transmission rate, we assessed the infection status of warm‐ and cool‐treated mothers and a subset of their offspring from each generation using dPCR and diagnostic PCR (Mackevicius‐Dubickaja et al. 2025). For each infected mother, transmission rates were calculated by dividing the number of infected offspring by the total number of offspring (Exp 1_*n*=4–8_; Exp 2_*n*=1–4_) tested per brood. We compared F2 transmission rates between warm and cool quintuply‐infected spider lines using a two‐tailed Fisher's Exact test (RTW123 = 39 lines). Diagnostic symbiont screening following experiments revealed that a subset of spider lines varied in their symbiotypes (RTW12 = 7 lines, RW123 = 1 line, RTW1 = 5 lines). We did not perform Fisher's Exact test to compare the transmission rates for these variant symbiotypes, as the lines either lacked a sufficiently large sample size for both temperatures or were only represented in the warm‐treated spider lines. Instead, we present the raw transmission rates for symbionts in these symbiotypes.

### 2.6 Titre Analysis

Symbiont titers were first normalised to a host *18S rRNA* gene as in Mackevicius‐Dubickaja et al. (2025). The symbiont titers across experimental groups (generation [P _*n*=21_, F1 _*n*=22_, F2 _*n*=20_, F3 _*n*=42_] and temperature treatment [Cool _*n*=57_ = 20°C; Warm _*n*=48_ = 28°C]) were analysed using the Kruskal‐Wallis test in JMP Pro (SAS Institute Inc.) statistical software. Post hoc pairwise comparisons were made using the Dunn method test to determine differences across generations and treatments.

### 2.7 Statistical Analysis of Symbiont Associations With Feminisation

To test associations between relative abundance (each symbiont titre divided by the sum of all symbiont titers) and feminisation status, we used Receiver Operating Characteristic (ROC) analysis to evaluate predictive strength (Area Under the Curve‐ AUC) and generalised linear models (GLM) with a binomial distribution. Analyses were performed in JMP Pro (SAS Institute Inc.) statistical software.

## 3 Results

### 3.1 Elevated Temperature Exposure Reduces Wolbachia 1‐Induced Feminisation

**FIGURE 2:**
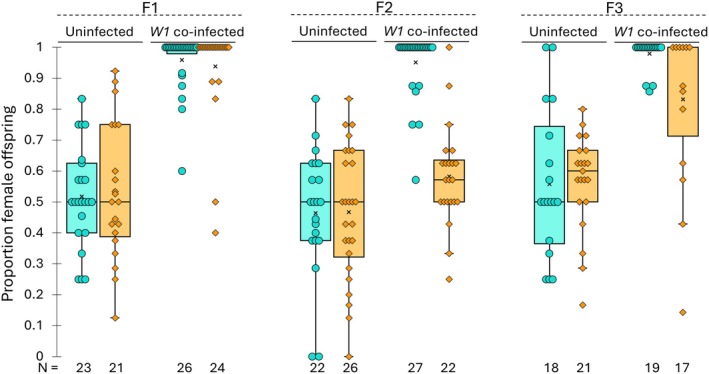
Effects of elevated F1 developmental temperature on feminisation across generations in *Mermessus fradeorum* . The proportion of female offspring in F1, F2, and F3 generations produced by uninfected and *Wolbachia 1* co‐infected females. Cool‐lines (teal) were continuously reared at 20°C, while Warm‐lines (orange) were exposed to elevated rearing temperature in the F1 generation. *N* below graphs indicate the number of broods per group.

Offspring sex ratio varied significantly across generations, infection group, and temperature treatment (ΔDeviance _Full model_ = 7.938, d.f. = 1, *p* = 0.024) (Figure 2). *Wolbachia 1* co‐infected F1 spider broods directly exposed to either warm or cool temperature treatments were significantly more female‐biased than uninfected spiders (ΔDeviance _Infect_ = 205.97, d.f. = 1, *p* < 0.0001). Warm temperatures did not alter F1 sex ratios (ΔDeviance _F1Tem*p*_ = 0.311, d.f. = 1, *p* = 0.681), regardless of their infection status (ΔDeviance _Temp * Infect_ = 0.875, d.f. = 1, *p* = 0.49).

Cool‐treated infected F2 broods were also significantly more feminised than uninfected F2 broods (ΔDeviance _Infect_ = 70.579, d.f. = 1, *p* < 0.0001). However, the F1 warm temperature treatment significantly affected the sex ratio of F2 infected offspring (ΔDeviance _F1Temp_ = 34.438, d.f. = 1, *p* < 0.0001). F1 infected spider exposure to warm temperatures resulted in feminisation failure in their F2 offspring (ΔDeviance _Temp * Infect_ = 42.429, d.f. = 1, *p* < 0.0001), producing a sex ratio resembling that of uninfected broods. Thus, despite not causing an immediate effect on the sex ratio of infected F1 broods, warm temperature had a delayed effect on the sex ratio of infected F2 broods.

As with the preceding generation, cool‐treated F3 infected broods retained their extreme female bias (ΔDeviance _Infect_ = 50.773, d.f. = 1, *p* < 0.0001), while warm developmental temperature in F1 significantly affected F3 infected offspring sex ratio (ΔDeviance _F1Temp_ = 14.205, d.f. = 1, *p* = 0.002). F3 descendants of warm‐treated infected spiders showed improved but imperfect feminisation: sex ratios were more female‐biased than warm‐treated F3 uninfected spiders, but less female‐biased than cool‐treated infected spiders (ΔDeviance _Temp * Infect_ = 29.514, d.f. = 1, *p* < 0.0001). This temperature‐induced reduction in feminisation rates persisted even after the removal of spider lines that had lost *Wolbachia 1* from the analysis (ΔDeviance _Temp * Infect_ = 17.434, d.f. = 1, *p* = 0.002), indicating that grandmaternal warm temperature exposure reduced *Wolbachia 1* feminisation rates in F3 spiders.

Variation in the proportion of female offspring in F3 descendants of warm‐treated spiders is associated with infection composition (Figure S1). F3 spider broods harbouring *Rickettsiella* and all three *Wolbachia* strains (W1, W2, and W3) exhibited complete feminisation (proportion female = 1.0, *n* = 4). Samples co‐infected with *Rickettsiella* and *Wolbachia* strain 1 and 2 also displayed high levels of feminisation (proportion female = 0.83–1, mea*n* = 0.94, *n* = 4). In contrast, individuals that retained only *Rickettsiella* and *Wolbachia 1* (the strain required for feminisation) showed greater variability in F3 sex ratios (proportion female = 0.14–1, mean = 0.77, *n* = 7). These results suggest that while *Wolbachia 1* is essential for feminisation, the presence of *Wolbachia 2* and *Wolbachia 3* improves phenotype recovery following grandparental (F1) exposure to elevated temperatures.

### 3.2 Elevated Temperatures Compromise Symbiont Transmission Rates

Symbiont transmission rates of cool‐treated spiders were generally perfect across generations, except for *Wolbachia 3*, which had a transmission rate of ~90% and ~92% in the F2 and F3 generations, respectively (Table S2). Following exposure to warm temperatures in F1, transmission of *Wolbachia 1* and *Wolbachia 2* to F2 offspring in co‐infected assemblies with all five symbionts was significantly reduced to ~76% and ~86% transmission, respectively (Table S2; Fisher's Exact Test W1 *p* < 0.0001; W2 *p* = 0.002). *Wolbachia 3* transmission rates to F2 offspring remained incomplete following exposure to warm temperatures (~82% transmission rate), although they did not differ significantly compared to transmission in cool‐treated broods (*p* = 0.278). *Wolbachia 1* transmission to F3 offspring remained perfect (100% infection rate) for a subset of spiders that retained all three *Wolbachia* strains following F1 heat treatment (Table S2). In contrast, *Wolbachia 1* transmission to F3 offspring remained incomplete (~88%) for spiders that originally lacked or lost *Wolbachia 2* and *Wolbachia 3* following warm temperature exposure (RW1), suggesting that the presence of multiple coinfecting *Wolbachia* strains may stabilise *Wolbachia 1* transmission. Exposure to warm temperatures in F1 also led to the complete loss of *Tisiphia* in F2 offspring. The *Tisiphia* transmission failure occurred in all warm‐treated spider lines, regardless of starting infection or experiment (Table S2). *Rickettsiella* transmission remained at perfect rates across both temperature treatments, all infection assemblies, and through each generation (Table S2).

### 3.3 Elevated Temperatures Induce Transgenerational Shifts in Symbiont Titre

**FIGURE 3:**
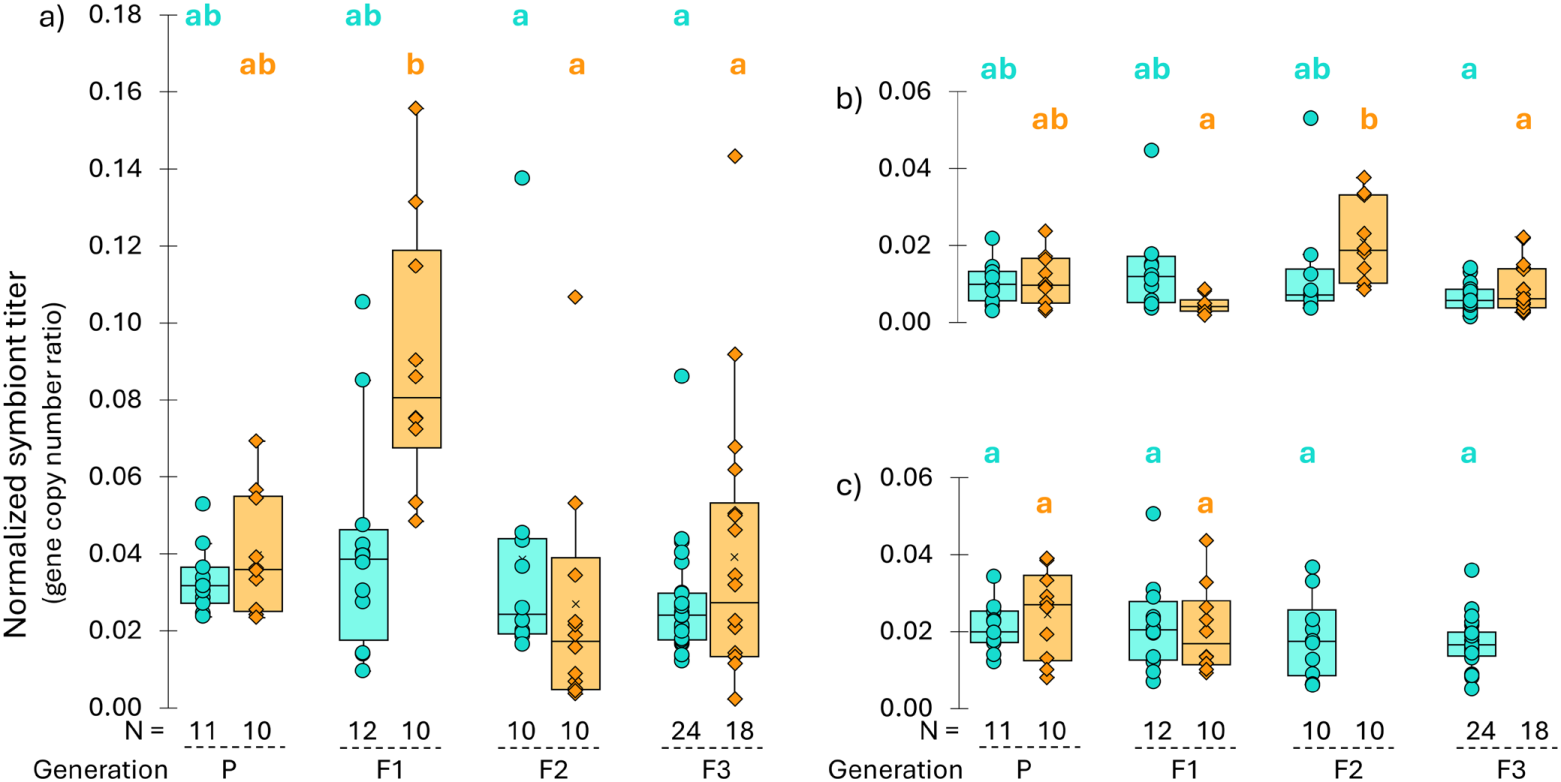
Effects of exposure to elevated temperature during ontogeny on *Wolbachia 1* (a), *Rickettsiella* (b), and *Tisiphia* (c) titers across generations in *Mermessus fradeorum* from experiment 2. Cool‐lines (teal) were continuously reared at 20°C, while Warm‐lines (orange) were exposed to a higher rearing temperature, 28°C, during F1 generation ontogeny. Following heat exposure (F1), *Tisiphia* was lost in all warm‐treated lines. Symbiont titers were normalised to the *M. fradeorum* *18S* rRNA gene. Generations (P‐F3) and sample size (N) are represented on the x‐axis. Kruskal‐Wallis tests were used for a full‐factorial comparison across generations (P‐F3) (a: H (7) = 28.24, *p* = 0.0002; b: H (7) = 30.57, *p* < 0.0001; c: H (5) = 5.59, *p* = 0.3482), followed by post hoc Dunn tests comparison between generations and treatment lines (indicated by letters above each graph, colour denotes F1 temperature treatment).

Cool‐treated spiders generally had stable symbiont titers throughout generations, whereas warm temperature exposure in F1 had a significant effect on symbiont titers (Figures 3 and S2). Warm temperature had an immediate effect on *Wolbachia 1* titre (Kruskal‐Wallis: H (7) = 28.24, *p* = 0.0002) (Figure 3a). Spiders reared under elevated F1 temperature conditions exhibited notably higher *Wolbachia 1* titers, with a mean titre of 0.09 (95% CI: 0.066, 0.115, *n* = 10). The increase observed in F1 was marginally different from the parental (P) generation (*p* = 0.1287) but significantly higher than in the F2 and F3 generations (*p* = 0.0004 and *p* = 0.0035, respectively). By F3, *Wolbachia 1* titers returned to baseline and did not differ from parental (P) lines (*p* = 1), indicating that the temperature effect on *Wolbachia 1* titre is transient and generation‐specific. In contrast, exposure to warm temperatures in F1 had an opposite and delayed effect on *Rickettsiella* titre (Kruskal‐Wallis: H (7) = 30.57, *p* < 0.0001) (Figure 3b). The F1 generation, which was reared under elevated temperatures, had the lowest *Rickettsiella* titre among all groups, with a mean titre of 0.005 (95% CI: 0.003, 0.006). However, it was only marginally different compared to the parental (P) generation (*p* = 0.0798). The reduced titre in F1 rebounded in F2 upon the return to 20°C, and offspring of the broods that experienced warm temperatures (F2) exhibited significantly higher *Rickettsiella* titers compared to their parents (F1) (*p* = 0.0002) and offspring (F3) (*p* = 0.0116). In F3, titers returned to baseline levels and were not significantly different compared to parental (P) titers (*p* = 1), indicating that the temperature effect on *Rickettsiella* is also transient. When present, *Tisiphia* titers remain stable across generations and treatments (Kruskal‐Wallis: H (5) = 5.59, *p* = 0.3482), although this apparent stability masks the underlying transmission failure following heat exposure (Figure 3c; Table S2). This indicates that *Tisiphia* is extremely sensitive to elevated temperature, though this sensitivity is not reflected in the titre when the symbiont is present.

Differences in *Wolbachia 2* and *Wolbachia 3* titers were not statistically significant (W2: Kruskal‐Wallis: H (7) = 7.71, *p* = 0.3589; W3: Kruskal‐Wallis: H (7) = 5.61, *p* = 0.5859) and stayed within a narrow range across generations (Figure S2).

### 3.4 Symbiont Quantity Dynamics Shape Reproductive Outcomes

**FIGURE 4:**
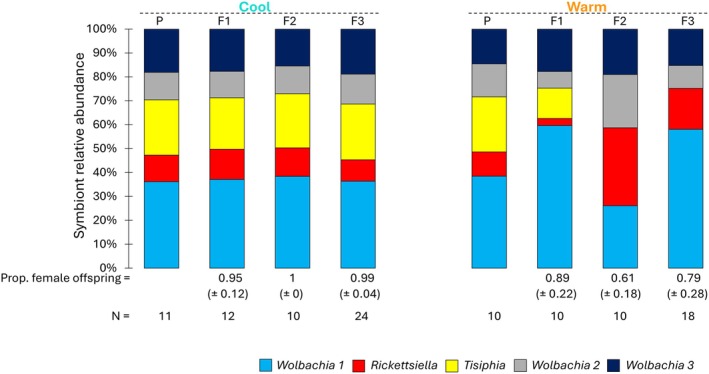
Effects of elevated developmental temperature on *Wolbachia 1* (light blue), *Rickettsiella* (red), *Tisiphia* (yellow), *Wolbachia 2* (grey), and *Wolbachia 3* (dark blue) relative abundance across generations in *Mermessus fradeorum* from experiment 2 in Cool‐ (left) and Warm‐lines (right). The proportion of female offspring in each generation and treatment is indicated at the bottom of the graph. Cool‐lines were continuously reared at 20°C, while Warm‐lines were exposed to a higher rearing temperature, 28°C, during F1 generation ontogeny. All infection assemblies were included. Sample size (*N*) is represented on the x‐axis.

To evaluate the temperature effect on community composition, we quantified the relative abundance of symbionts, defined as their proportion of the total symbiont titre within the community. The consistently female‐biased *Wolbachia 1* co‐infected spider broods from the cool lines exhibited stable symbiont relative abundance through the generations, with *Wolbachia 1* representing the largest portion of the symbiont community (Figure 4a). In contrast, warm temperature exposure in the F1 generation significantly affected symbiont relative abundance across generations, with this altered abundance also correlating with shifts in feminisation rate (Figure 4b).

In warm‐treated spiders, maternal (F1) *Wolbachia 1* relative abundance was significantly negatively associated with feminisation rate in offspring (F2) [GLM: L‐R $\chi^{2}$ = 7.52, *p* = 0.0061], but was a weak predictor of feminisation via ROC analysis [AUC = 0.659, 95% CI: 0.471–0.847]. This suggests that a higher relative abundance of *Wolbachia 1* in heat‐treated adult mothers (F1) is associated with a lower likelihood of offspring (F2) feminisation. Heat‐reduced feminisation rates of offspring (F2) were also significantly negatively associated with *Rickettsiella* relative abundance in those offspring [GLM: L‐R $\chi^{2}$ = 11.21, *p* = 0.0008]. Decrease in *Rickettsiella* relative abundance moderately predicts brood feminisation rate via ROC analysis [AUC = 0.748, 95% CI: 0.545–0.952], linking high *Rickettsiella* relative abundance with reduced feminisation rates within the same generation.

Given that these patterns suggest a potential interaction between symbionts, we next tested whether the ratio between *Wolbachia 1* and *Rickettsiella* better explains variation in feminisation rate. The *Wolbachia 1* to *Rickettsiella* ratio was positively associated with offspring feminisation [GLM: β = 0.96 ± 0.29 SE, *z* = 3.34, *p* = 0.0008]. However, this association was primarily driven by a strong negative association between *Rickettsiella* relative abundance and the proportion of female offspring [GLM: β = −5.33 ± 1.4 SE, *t* = −3.81, *p* = 0.0001], whereas *Wolbachia 1* relative abundance was not a significant predictor [GLM: β = −0.38 ± 1.91 SE, *t* = −0.2, *p* = 0.84]. Together, these results suggest that *Rickettsiella* plays an inhibitory role in the feminisation process, with the feminisation rate primarily driven by variation in its relative abundance.

Variation in symbiont relative abundance across generations in warm lines was also linked to symbiont community composition (Figure S3). Spider broods harbouring all three *Wolbachia* strains (W1, W2, and W3) maintained more stable relative abundances across all four generations, with *Wolbachia 1* consistently representing the largest portion of the symbiont community (P—F3). Despite the loss of *Tisiphia* following heat exposure, the relative abundance of *Rickettsiella* and *Wolbachia 1* in warm‐treated RW123 lines remained within 5%–15% and 39%–62% (*n* = 10) across generations, respectively. In contrast, in symbiotype communities lacking the W2 and/or W3 strains, the relative abundance of *Wolbachia 1* and *Rickettsiella* exhibited pronounced fluctuations following exposure to warm temperatures. In assemblies lacking *Wolbachia 3* (RTW12), *Rickettsiella* and *Wolbachia 1* relative abundances ranged from 5%–54% and 12%–68% (*n* = 6), respectively, while in broods lacking both *Wolbachia 2* and *Wolbachia 3* (RTW1), these ranges expanded to 3%–83% and 17%–87% (*n* = 32), respectively. These results suggest that the presence of *Wolbachia 2* and *Wolbachia 3* enhances the stability and resilience of symbiont dynamics under thermal stress. However, fluctuations in the relative abundance of *Wolbachia 2* and *Wolbachia 3* themselves were not associated with changes in feminisation rate (Figure 4).

## 4 Discussion

In this study, we exposed *Mermessus fradeorum* spiders to warm temperatures during post‐embryonic development for a single generation and followed its effect on feminisation and symbiont dynamics in adults across subsequent generations. We found that warm temperatures did not immediately affect the feminisation of treated spiders (F1), but significantly reduced feminisation in the next generation (F2), with a weaker but persistent effect in the following generation (F3) (Figure 2). Exposure to warm temperatures also affected symbiont transmission from the F1 to F2 generation (Table S2). *Wolbachia 1* and *Wolbachia 2* transmission in quintuple‐infected individuals was significantly reduced, and *Tisiphia* transmission to F2 offspring completely failed across all symbiont community assemblies. We also observed an immediate increase in *Wolbachia 1* titre in the heat‐exposed generation (F1) and a delayed *Rickettsiella* titre increase in their offspring (F2) (Figure 3). These quantitative shifts resulted in altered symbiont relative abundances and variation in feminisation outcomes (Figure 4): Increased maternal *Wolbachia 1* relative abundance in F1 was associated with reduced feminisation in F2, while non‐feminised offspring in F2 exhibited elevated relative *Rickettsiella* abundance.

Direct heat exposure during post‐embryonic development did not affect feminisation in the exposed generation (F1). However, the offspring from successive generations (F2 and F3) were significantly influenced by F1 exposure, showing reduced feminisation rates. The cause for this delayed feminisation failure is unclear but may reflect the timing of feminisation induction in *M. fradeorum* . The onset of *Wolbachia*‐induced feminisation occurs throughout juvenile development in various host taxa. In the leafhopper *Zyginidia pullulan*, *Wolbachia* modifies sexual differentiation during nymphal stages (Negri et al. 2009); in isopods, feminisation is induced during early juvenile stages (Herran et al. 2020; Badawi et al. 2015); and in *Eurema hecabe* butterflies, it is induced during larval development and requires the constant presence of *Wolbachia* (Narita et al. 2007). However, in *M. fradeorum* , exposure to elevated temperatures during juvenile development did not immediately disrupt feminisation, suggesting that feminisation by *Wolbachia 1* is induced before spiderlings leave the egg mass, possibly during embryogenesis or maternal oogenesis. The delayed inhibition of feminisation in generations F2 and F3 also suggests a transgenerational temperature effect on the symbionts and/or the host regulatory mechanisms required for feminisation. Similar transgenerational effects have been observed in other systems: for example, heat stress reduces *Wolbachia* density and maternal transmission in *Aedes aegypti* mosquitoes, affecting reproduction in subsequent generations (Foo et al. 2019). In *Drosophila hydei* , rearing at a lower temperature reduces *Spiroplasma* titre, disrupts vertical transmission, and reduces protection against wasp parasitism even upon return to warmer conditions (Corbin et al. 2021).

The mechanism underlying *Wolbachia 1*‐induced feminisation in *M. fradeorum* remains unresolved, but our findings suggest that the quantitative interaction between *Wolbachia 1* and *Rickettsiella* plays a key role in shaping offspring sex ratios (Figure 4). Prior work found no correlation between maternal *Wolbachia 1* titre and feminisation rate (Mackevicius‐Dubickaja et al. 2025). That study, however, was conducted under stable, permissive environmental conditions, where feminisation rate remained consistently high and symbiont titers were comparable to those observed in hosts reared consistently at 20°C in the present study. Our current results reveal that symbiont relative abundance is associated with reproductive phenotypes. We observed a consistent inverse relationship between *Wolbachia 1* and *Rickettsiella* relative abundance, aligning with shifts in offspring feminisation rate. High feminisation rates coincided with lower *Rickettsiella* relative abundance, whereas reduced feminisation was observed when *Rickettsiella* became more dominant. These patterns are suggestive of an antagonistic interaction between *Rickettsiella* and *Wolbachia 1*, where *Rickettsiella* may suppress or interfere with *Wolbachia 1*‐mediated feminisation. This is supported by the finding that *Rickettsiella* relative abundance in offspring was a strong negative predictor of the feminised offspring. Feminisation appears to require not only the presence of *Wolbachia 1* but also its relative dominance over *Rickettsiella*, suggesting a competitive relationship between them. Similar antagonism has been reported in *Tetranychus turkestani*, where *Rickettsia* disrupted *Wolbachia*‐induced cytoplasmic incompatibility (Wang et al. 2025).

Feminisation rates are also correlated with the shift in symbiont community compositions, particularly with the number of *Wolbachia* strains present (Figure S1). In the F3 generation of warm‐treated individuals, spiders that retained only *Rickettsiella* and the primary feminising strain, W1, exhibited the greatest variability in sex ratios, while spiders additionally co‐infected with *Wolbachia* 2 had consistently high feminisation. Complete feminisation in the F3 generation occurred in individuals retaining all three *Wolbachia* strains. These patterns suggest that while *Wolbachia 2* and *Wolbachia 3* do not directly feminise *M. fradeorum* , their presence may stabilise or enhance the feminising effect of *Wolbachia 1*, perhaps by mediating *Wolbachia 1—Rickettsiella* interactions (Mackevicius‐Dubickaja et al. 2025). This stabilising effect may buffer against thermal disruptions to symbiont function and transmission as suggested in Mackevicius‐Dubickaja et al. (2025). Additionally, spider broods harbouring all three *Wolbachia* strains maintained relatively stable symbiont abundances across generations, despite the loss of *Tisiphia* in F2 (Figure 4), suggesting that co‐infection by *Wolbachia 2* and *Wolbachia 3* promotes stable partitioning of symbiont abundance within the symbiont community. Similar synergistic relationships have been documented in other systems. For example, in aphids, the obligate symbiont *Buchnera* recovers more effectively from heat stress when co‐infecting facultative symbionts *Regiella* or *Fukatsuia* are present, enhancing host fitness under environmental stress (Heyworth et al. 2020). Aphids co‐infected with *Hamiltonella* and *Serratia symbiotica* also exhibit greater resistance to parasitoid wasps compared to singly infected individuals (McLean et al. 2018). Likewise, *Hylyphantes graminicola* spiders co‐infected with *Wolbachia* and *Cardinium* demonstrate enhanced fat and amino acids synthesis, impacting host metabolism (Li et al. 2020).

Symbiont titre often correlates with transmission success, with reduced titers resulting in transmission failure (Mancini et al. 2021; Hurst et al. 2001). However, symbiont titre had more variable consequences for transmission rate in *M. fradeorum*, and titers generally did not correspond with symbiont transmission rates following heat exposure. *Wolbachia 1* titre surprisingly increased following heat treatment, yet *Wolbachia 1* also suffered reduced transmission rates. In contrast, heat moderately reduced *Rickettsiella* titre, consistent with previous results linking warmer conditions to decreased *Rickettsiella* titre in *M. fradeorum* (Proctor et al. 2024). This reduction in *Rickettsiella* titre did not reduce transmission rate, which remained perfect at warm temperatures. Thermally robust transmission rates potentially allow *Rickettsiella* to withstand bouts of warm temperatures in nature, which may help explain why *Rickettsiella* is near fixation in field‐collected *M. fradeorum* populations (Rosenwald 2020; Rosenwald et al. 2020). In further contrast, the symbiont *Tisiphia* exhibited complete transmission failure following heat exposure despite showing no titre change in heat‐treated *M. fradeorum* spiders. The causes underlying these discrepancies between titre and transmission rate remain unclear, although elevated temperatures may variably affect the viability or functional integrity of symbiont cells or alter their localisation within developing oocytes, the host tissue directly relevant to maternal transovarial symbiont transmission. Symbiont titre and transmission could also be differentially affected by symbiont and/or host stress responses, leading to a potential disconnect between titre and transmission success. Alternatively, the *Wolbachia 1* and *Tisiphia* titers in the heat‐treated F1 generation may be inflated due to the presence of residual DNA from dead bacteria if exposure to 28°C was lethal.

The five symbionts of *M. fradeorum* exhibit considerable variation in their temperature sensitivity, with *Wolbachia* and *Tisiphia* suffering transmission failure at warm temperatures. These symbionts also occur at lower frequencies compared to *Rickettsiella* in natural *M. fradeorum* populations, suggesting that temperature sensitivity may specifically impede the spread of these symbionts (Rosenwald et al. 2020). *Wolbachia 1* temperature sensitivity was mitigated by co‐infection with additional *Wolbachia* strains, which stabilised *Wolbachia* transmission rates and improved feminisation recovery following warm temperature exposure. Co‐infection also enhances *Wolbachia* feminisation at benign temperatures (Mackevicius‐Dubickaja et al. 2025). These synergistic effects of co‐infection may promote the spread of feminising *Wolbachia 1* as a five‐member symbiont consortium, given that *Wolbachia 1* is most commonly found as a co‐infection with all five symbionts in *M. fradeorum* populations (Rosenwald 2020). Additionally, while an earlier study demonstrated that *Rickettsiella* titers dropped sharply under heat in singly infected spiders (Proctor et al. 2024), here we observed only a mild decrease in maternal titre and a notable rebound in offspring titre when *Rickettsiella* was part of a multi‐symbiont community. This contrast suggests that co‐infection also stabilises *Rickettsiella* titre under heat stress. These findings indicate that the presence of all three *Wolbachia* strains plays a pivotal role not only in symbiont transmission and phenotype expression but also in shaping the stability and composition of the symbiont community.

While these results reveal significant patterns, several features of the study design may constrain their interpretation. First, combining the data of two experiments conducted under different temperature regimes may affect the extent and timing of symbiont transmission failure and feminisation rate. Despite this, the observed phenotypic patterns were consistent, supporting a robust temperature effect across the two regimes. Second, symbiont quantification was restricted to a subset of individuals from Experiment 2, limiting generalisation to the full dataset; nevertheless, this subset captured the full range of phenotypic variation, providing good proximity into observed symbiont‐feminisation associations. Third, small sample sizes for some broods may have limited the detection of subtle effects; however, these cases still reveal the natural variability of symbiont communities to provide valuable insights. Fourth, the uneven generational design between experiments may also have influenced the detection of temperature effects across generations, though the similar trends observed in the overlapping generations (P‐F2) indicate that the main patterns are unlikely to be experiment‐specific. Finally, the observed associations between *Wolbachia 1*, *Rickettsiella*, and feminisation rate are correlative; while the patterns suggest inhibitory interactions, causal relationships are yet to be experimentally tested.

Despite these limitations, this study demonstrates how environmental history can shape the evolutionary stability of microbial‐host systems. In natural populations, where spider hosts experience daily and seasonal thermal fluctuations, such environmentally driven shifts in symbiont communities are likely to influence transmission efficiency and the expression of symbiont‐induced phenotypes, with consequences for host population structure and sex ratio dynamics. Occasional reductions in feminisation rates may be important for maintaining males within the population and avoiding demographic collapse. However, the role of the male symbiont community, as well as the effect of prolonged or repeated thermal stress in this system is yet unknown. Past seasonal and daily thermal variation will need to be considered when exploring arthropod and symbiont responses to environmental variables.

## Author Contributions

All authors designed the experiment. V.M.‐D., E.E.W., and M.R.D. conducted the experiments. V.M.‐D., E.K., and M.R.D. conducted statistical analyses and generated figures. J.A.W. and Y.G. supervised the project. All authors contributed to the manuscript and approved the final version.

## Funding

This work was supported by the National Science Foundation (Grant 1953223), United States ‐ Israel, Binational Science Foundation (BSF) (Grant 201697), National Institute of Food and Agriculture (Grants 1020740, 7007679, 2023‐67012‐39352) and Israel Science Foundation (Grant 2809/23).

## Conflicts of Interest

The authors declare no conflicts of interest.

## Supporting information


**Figure S1:** Proportion of female offspring across generations under elevated F1 developmental temperature in *Mermessus fradeorum* . Female spiders in F1 were reared at elevated temperature, starting from three infection assemblies: all five symbionts (*Rickettsiella*, *Tisiphia*, and three *Wolbachia* strains; W1‐3), four (*Rickettsiella*, *Tisiphia*, and two *Wolbachia* strains; W1, W2), or three (*Rickettsiella*, *Tisiphia*, and *Wolbachia* strain 1). Elevated temperature led to loss of *Tisiphia*, so that in F2 and F3 generations these assemblies shifted to four, three, and two symbionts, respectively. Pie charts indicate parental infection assemblies, and N below the graph indicates the sample size per group.
**Figure S2:** Effects of exposure to elevated temperature during ontogeny on *Wolbachia* 2 (a) and *Wolbachia* 3 (b) titers across generations in *Mermessus fradeorum* from experiment 2. Cool‐lines (teal) were continuously reared at 20°C, while Warm‐lines (orange) were exposed to a higher rearing temperature, 28°C, during F1 generation ontogeny. Symbiont titers were normalised to the *M. fradeorum* 18S rRNA gene. Generations (P‐F3) and sample size (N) are represented on the x‐axis. Kruskal‐Wallis tests were used for a full‐factorial comparison across generations (P‐F3) and treatments (cool and warm) (a: H (7) = 7.71, *p* = 0.3589; b: H (7) = 5.61, *p* = 0.5859).
**Figure S3:** *Wolbachia* 1 (a), *Rickettsiella* (b), and *Tisiphia* (c) relative abundance across generations under elevated F1 developmental temperature in *Mermessus fradeorum* from experiment 2. Female spiders in F1 were reared at an elevated temperature, starting from three infection assemblies: RTW123—dark (deep) green (*Rickettsiella*, *Tisiphia*, and three *Wolbachia* strains; W1‐3); RTW12—medium (vivid) green (*Rickettsiella*, *Tisiphia*, and two *Wolbachia* strains; W1, W2); RTW1—light (pale) green (*Rickettsiella*, *Tisiphia*, and *Wolbachia* strain 1). Elevated temperature led to loss of *Tisiphia*, so that in F2 and F3 generations these assemblies shifted to four (RW123), three (RW12), and two (RW1) symbionts, respectively. Generations (P‐F3) and sample size (N) are represented on the x‐axis.
**Table S1:** Primer pairs and probes used in this study, with references provided for previously published primers for each symbiont used in diagnostic and digital PCR.
**Table S2:** Effects of exposure to elevated temperature during ontogeny on symbiont transmission across generations in *Mermessus fradeorum* . Cool‐lines (teal) were continuously reared at 20°C, while Warm‐lines (orange) were exposed to an elevated temperature during F1 generation ontogeny. The column on the left indicates symbionts present in parental (P) lines. The diagnostic and dPCR were used to assess the infection status of warm‐ and cool‐treated mothers and a subset of their offspring from each generation. Transmission rates were calculated by dividing the number of infected offspring by the total number of offspring tested per brood.

**Data S1.** Symbiont infection is represented by a letter to signify genus and number for strain: *R* = *Rickettsiella*, T = *Tisiphia*, W = *Wolbachia* 1–3. Uninfected spiders did not harbour symbionts. The sample name is composed of a letter and a number indicating generation (P, F1, F2, F3), infection status (I—infected; U—uninfected), sample number (Sn), and eggmass used for treatment line (a: cool‐line; b: warm‐line). See details in the Materials and Methods section.
Proportion of female offspring (tab 1): To evaluate the proportion of female offspring in two independent experiments (Experiment 1 and Experiment 2), we recorded the sex ratio of offspring at adulthood by identifying the sex of 4–26 randomly selected offspring per mother.
Transmission rate (tab 2): A subset of samples from both experiments was used for diagnostic PCR to evaluate symbiont transmission rate.
Digital PCR (tab 3): A subset of individuals from Experiment 2 was used for evaluation symbiont transmission rate and symbiont quantification using dPCR.

## Data Availability

All data associated with this manuscript are available in the Supporting Information.
