# Supplementary material for: Elevated Temperatures Disrupt Wolbachia‐Induced Feminisation and Reshape Microbial Community Dynamics Across Generations in a Spider Host
Source: Mol Ecol. 2026 May 8;35:e70371. doi: 10.1111/mec.70371 (PMC13156531; doi:10.1111/mec.70371)
Supplement: Supplementary file 1 — Figure S1: Proportion of female offspring across generations under elevated F1 developmental temperature in Mermessus fradeorum . Female spiders in F1 were reared at elevated temperature, starting from three infection assemblies: all five symbionts (Rickettsiella, Tisiphia, and three Wolbachia strains; W1‐3), four (Rickettsiella, Tisiphia, and two Wolbachia strains; W1, W2), or three (Rickettsiella, Tisiphia, and Wolbachia strain 1). Elevated temperature led to loss of Tisiphia, so that in F2 and F3 generations these assemblies shifted to four, three, and two symbionts, respectively. Pie charts indicate parental infection assemblies, and N below the graph indicates the sample size per group. Figure S2: Effects of exposure to elevated temperature during ontogeny on Wolbachia 2 (a) and Wolbachia 3 (b) titers across generations in Mermessus fradeorum from experiment 2. Cool‐lines (teal) were continuously reared at 20°C, while Warm‐lines (orange) were exposed to a higher rearing temperature, 28°C, during F1 generation ontogeny. Symbiont titers were normalised to the M. fradeorum 18S rRNA gene. Generations (P‐F3) and sample size (N) are represented on the x‐axis. Kruskal‐Wallis tests were used for a full‐factorial comparison across generations (P‐F3) and treatments (cool and warm) (a: H (7) = 7.71, p = 0.3589; b: H (7) = 5.61, p = 0.5859). Figure S3: Wolbachia 1 (a), Rickettsiella (b), and Tisiphia (c) relative abundance across generations under elevated F1 developmental temperature in Mermessus fradeorum from experiment 2. Female spiders in F1 were reared at an elevated temperature, starting from three infection assemblies: RTW123—dark (deep) green (Rickettsiella, Tisiphia, and three Wolbachia strains; W1‐3); RTW12—medium (vivid) green (Rickettsiella, Tisiphia, and two Wolbachia strains; W1, W2); RTW1—light (pale) green (Rickettsiella, Tisiphia, and Wolbachia strain 1). Elevated temperature led to loss of Tisiphia, so that in F2 and F3 generations these assemblies shift [file MEC-35-e70371-s001.docx]

Running title: Heat alters transgenerational symbiont dynamics

Title: Elevated temperatures disrupt *Wolbachia*-induced feminization and reshape microbial community dynamics across generations in a spider host

Supplementary Material

Virginija Mackevicius-Dubickaja^1^, Jennifer A. White^2^, Ellen E. Williams^2^, Eyal Klement^1^, Yuval Gottlieb^1*^, Matthew R. Doremus^3*^

^1^ Koret School of Veterinary Medicine, The Robert H. Smith Faculty of Agriculture, Food and Environment, The Hebrew University, Rehovot, Israel.

^2^ Department of Entomology, University of Kentucky, Lexington, KY, United States.

^3^ Department of Entomology, University of Illinois at Urbana-Champaign, Urbana, IL, United States

*To whom correspondence should be addressed.

email: [gottlieb.yuval@mail.huji.ac.il](mailto:gottlieb.yuval@mail.huji.ac.il); mdoremus@illinois.edu

Contents
Fig S1. Effects of heat exposure on feminization rate per infection assembly across generations

Fig S2. Variation in *Wolbachia 2* and *Wolbachia 3* titers across generations

Fig S3. Variation in *Wolbachia 1*, *Rickettsiella*, and *Tisiphia* relative abundance per infection assembly across generations

Table S1. Primer pairs and probes used in this study

Table S2. Effects of heat exposure on symbiont transmission across generations


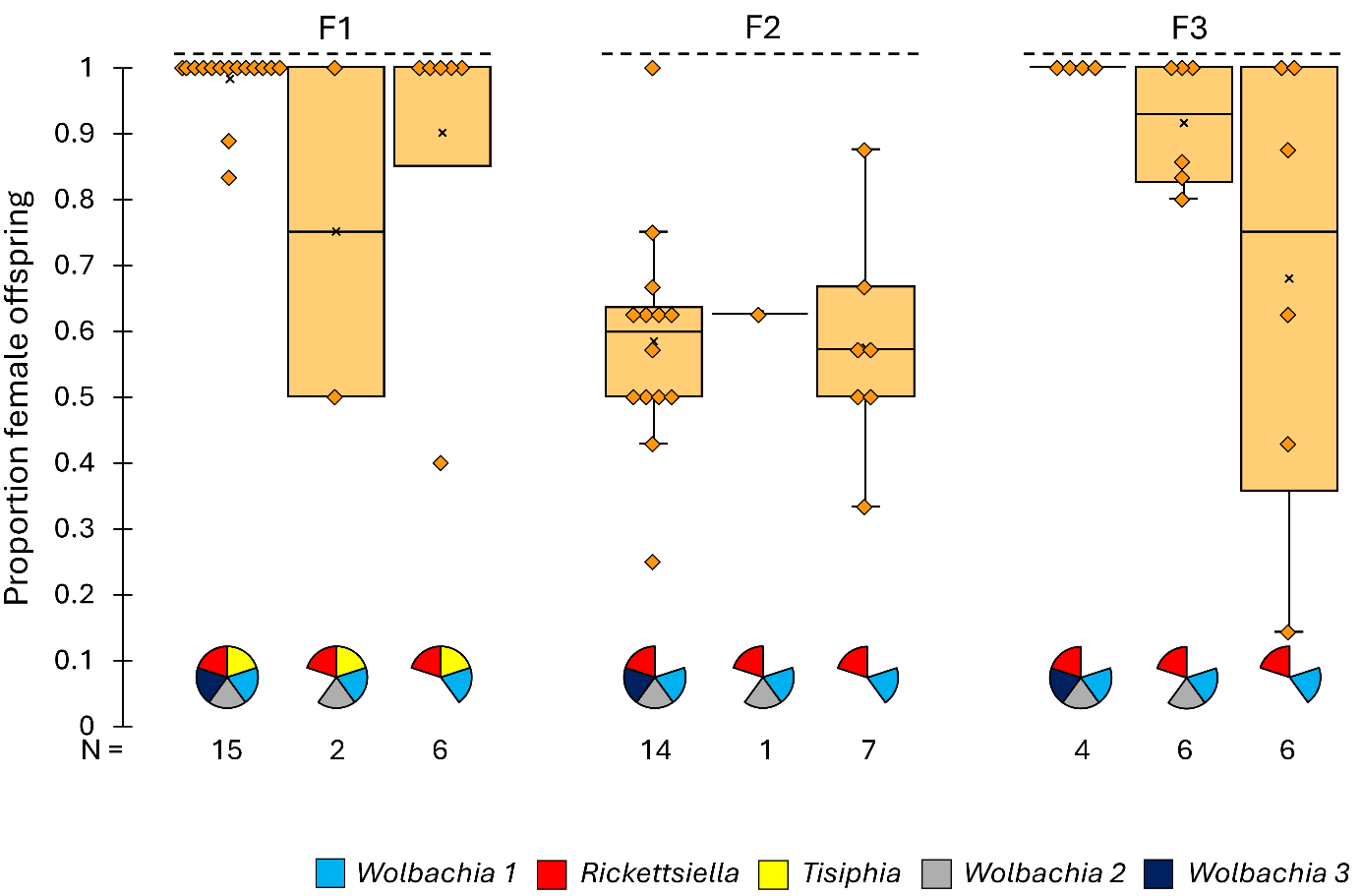


Figure S1 Proportion of female offspring across generations under elevated F1 developmental temperature in Mermessus fradeorum. Female spiders in F1 were reared at elevated temperature, starting from three infection assemblies: all five symbionts (Rickettsiella, Tisiphia, and three Wolbachia strains; W1-3), four (Rickettsiella, Tisiphia, and two Wolbachia strains; W1, W2), or three (Rickettsiella, Tisiphia, and Wolbachia strain 1). Elevated temperature led to loss of Tisiphia, so that in F2 and F3 generations these assemblies shifted to four, three, and two symbionts, respectively. Pie charts indicate parental infection assemblies, and N below the graph indicates the sample size per group.


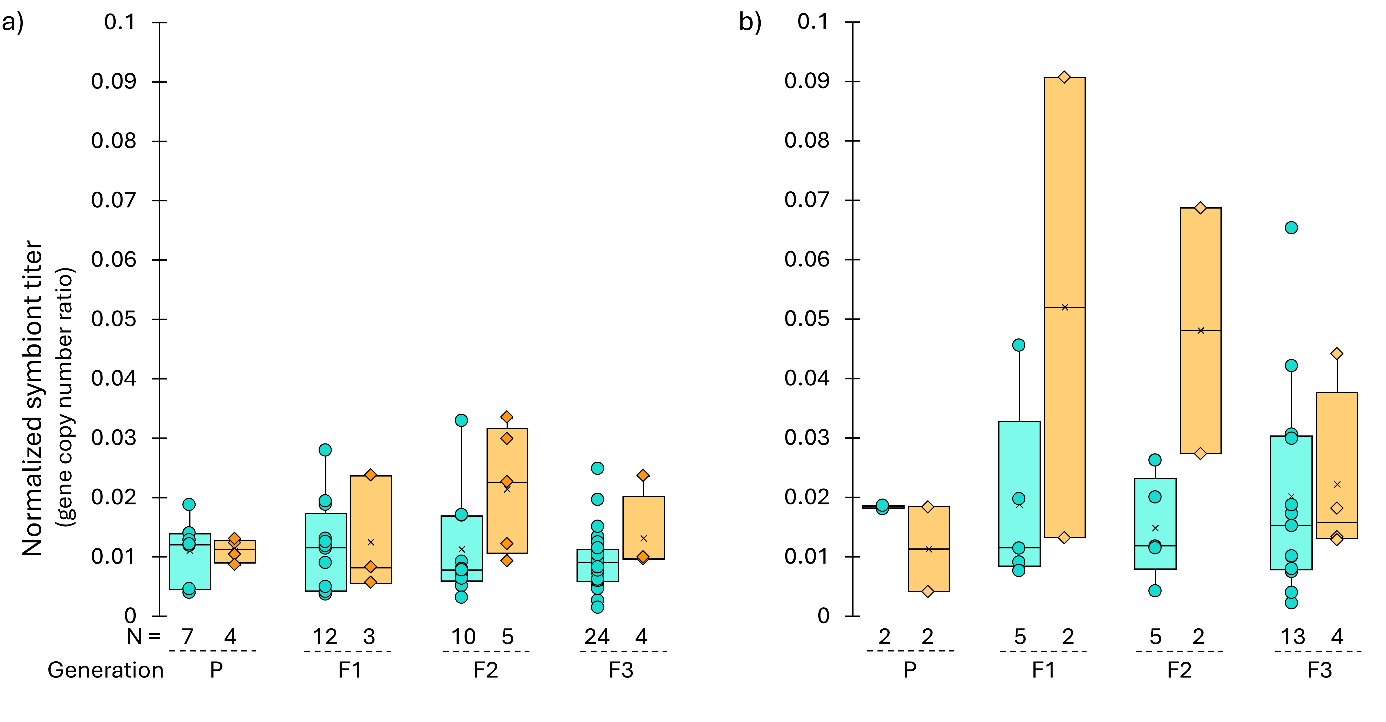


Figure S2 Effects of exposure to elevated temperature during ontogeny on Wolbachia 2 (a) and Wolbachia 3 (b) titers across generations in Mermessus fradeorum from experiment 2. Cool-lines (teal) were continuously reared at 20°C, while Warm-lines (orange) were exposed to a higher rearing temperature, 28°C, during F1 generation ontogeny. Symbiont titers were normalized to the M. fradeorum 18S rRNA gene. Generations (P-F3) and sample size (N) are represented on the x-axis. Kruskal-Wallis tests were used for a full-factorial comparison across generations (P-F3) and treatments (cool and warm) (a: H (7) = 7.71, p = 0.3589; b: H (7) = 5.61, p = 0.5859).


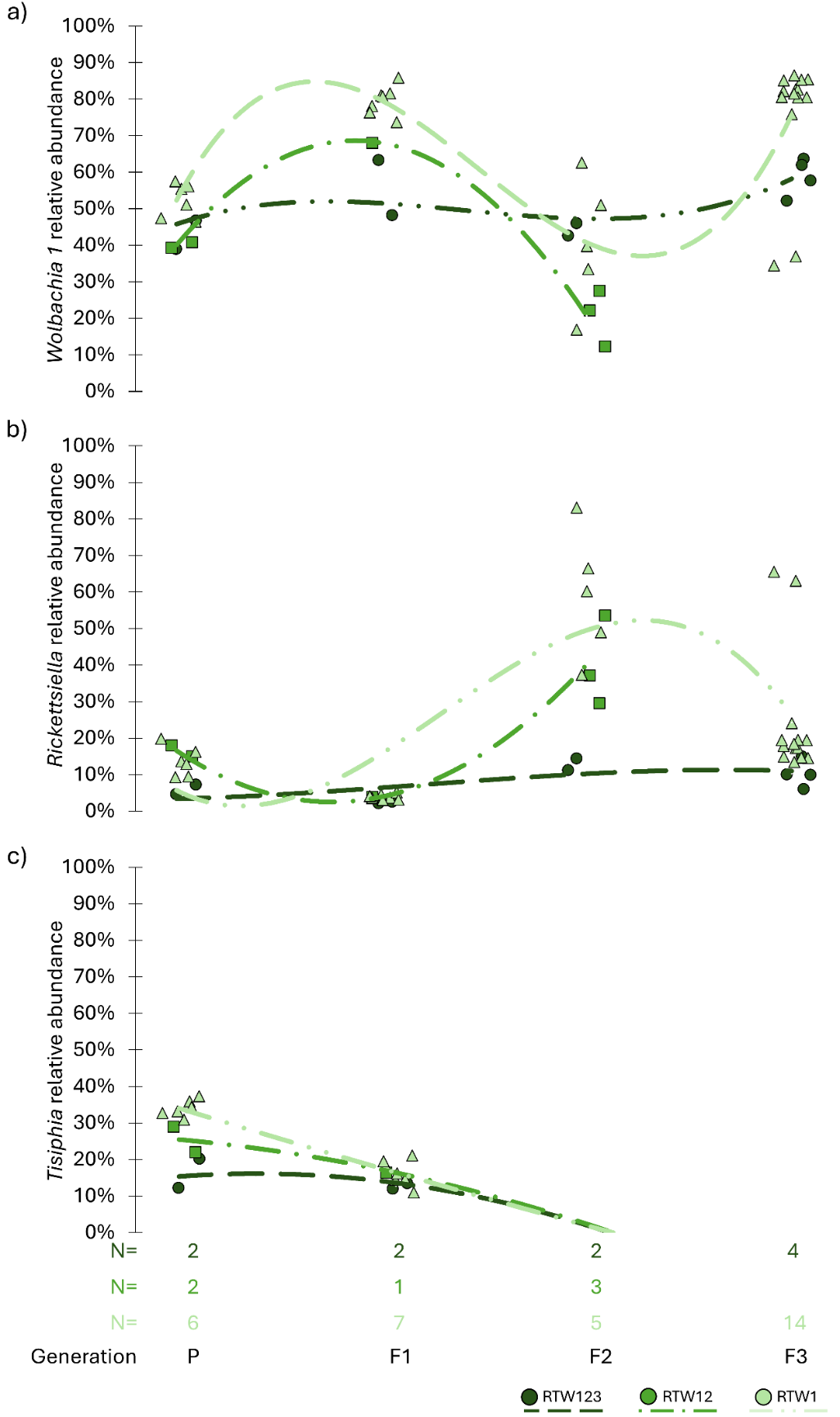


Figure S3 Wolbachia 1 (a), Rickettsiella (b), and Tisiphia (c) relative abundance across generations under elevated F1 developmental temperature in Mermessus fradeorum from experiment 2. Female spiders in F1 were reared at an elevated temperature, starting from three infection assemblies: RTW123 – dark (deep) green (Rickettsiella, Tisiphia, and three Wolbachia strains; W1-3); RTW12 – medium (vivid) green (Rickettsiella, Tisiphia, and two Wolbachia strains; W1, W2); RTW1 – light (pale) green (Rickettsiella, Tisiphia, and Wolbachia strain 1). Elevated temperature led to loss of Tisiphia, so that in F2 and F3 generations these assemblies shifted to four (RW123), three (RW12), and two (RW1) symbionts, respectively. Generations (P-F3) and sample size (N) are represented on the x-axis.

Table S1 Primer pairs and probes used in this study, with references provided for previously published primers for each symbiont used in diagnostic and digital PCR.

|  | **Target** | **Target gene** | **Primer/probe name** | **Sequence 5’to 3’** | **Amplicon length (bp)** | **Citation** |
| --- | --- | --- | --- | --- | --- | --- |
| Digital PCR | *M. fradeorum* | *18S rRNA* | 18S_1375F | 5’-CATGGAGCTTGCGGTTCAAT-3’ | 170 | Mackevicius-Dubickaja, et al., 2025 |
|  |  |  | 18S_1544R | 5’-AGAGCCTCGTCCGTTATCAG-3’ |  |  |
|  |  |  | 18S_P | 5’-FAM-TCCAGGCCAGGACACAGGGAGGATT-BHQ1-3’ |  |  |
|  | *Rickettsiella* | *recA* | recA_612F | 5’-GGAAACAACAACGGGTGGTA-3 | 120 | Mackevicius-Dubickaja, et al., 2025 |
|  |  |  | recA_811R | 5’-CACCGAGTCGACAAATACCC-3’ |  |  |
|  |  |  | recA_P | 5’-ROX-ACGCGCTCAAGTTTTATGCTTCCGTACGT-BHQ2-3’ |  |  |
|  | *Tisiphia* | *rpoB* | rpoB_813F | 5’-AGCCTTGATGGGGTCAAACA-3’ | 121 | Mackevicius-Dubickaja, et al., 2025 |
|  |  |  | rpoB_933R | 5’-GGCTACAACTGAAACCCCAGA-3’ |  |  |
|  |  |  | rpoB_P | 5’-Atto550-TGCAACGTCAGGCTGTCCCGCTT-BHQ2-3’ |  |  |
|  | *Wolbachia1* | *wsp1* | wsp1_P | *5’-HEX-AATGATACCAATGCTGCAGATGGTG-BHQ1-3’ | 138 | Mackevicius-Dubickaja, et al., 2025 |
|  |  |  | wsp1/wsp2_F | 5’-TGATGTTGAAGGGCTTTACTCACA-3’ |  |  |
|  | *Wolbachia2* | *wsp2* | wsp1/wsp2_R | 5’-GGCATATCTTCAATCGCTACATCG-3’ |  |  |
|  |  |  | wsp2_P | *5’-Cy5-GCTGCTGAGACAAATGTTGCAGATA-BHQ2-3’ |  |  |
|  | *Wolbachia3* | *wsp3* | wsp3_209F | 5’-GCCTATCACTCCATACGTTGGT-3’ | 118 | Mackevicius-Dubickaja, et al., 2025 |
|  |  |  | wsp3_326R | 5’-ACCAGCTTTTACTTGACCAGCAA-3’ |  |  |
|  |  |  | wsp3_P | *5’-FAM-AACCGCTGTGAATGATCAAAACAGT-BHQ1-3’ |  |  |
| Diagnostic PCR | *Rickettsiella* | *16S rRNA* | RLA16s F1 | CAGTAAARRTTTCGGYCTTTAYGGG | 532 | Duron, et al., 2016 |
|  |  |  | RLA16s R1 | CGTGTAGGTGGTTGACTAGGTTTG |  |  |
|  | *Tisiphia* | *16S rRNA* | RicklongF | ACGTGGGAATCTACCCATCA | 530 | Curry, et al., 2015 |
|  |  |  | RicklongR | TAGCCTAGATGACCGCCTTC |  |  |
|  | *Wolbachia1* | *wsp* | wsp1_36F | ACAAAAGCATCAGGTCAAGAAAAT | 167 | Mackevicius-Dubickaja, et al., 2025 |
|  |  |  | wsp1_201R | CATCTGCAGCATTGGTATCATTT |  |  |
|  | *Wolbachia2* | *wsp* | Wsp2_93F | GCAAGGCAACAAATAAAGACAAGG | 163 | Mackevicius-Dubickaja, et al., 2025 |
|  |  |  | wsp2_255R | ACATTTGTCTCAGCAGCAGC |  |  |
|  | *Wolbachia3* | *wsp* | wsp3_270F | ACCGCTGTGAATGATCAAAACA | 162 | Mackevicius-Dubickaja, et al., 2025 |
|  |  |  | 2sp3_431R | ATCGTTATTAGTTGATGTTGTTGCTT |  |  |

# References

Curry, M. M. et al., 2015. Multiple endosymbiont infections and reproductive manipulations in a linyphiid spider population. *Heredity (Edinb),* 115(2), pp. 146-152, doi:10.1038/hdy.2015.2.

Duron, O., Cremaschi, J. & McCoy, K. D., 2016. The High Diversity and Global Distribution of the Intracellular Bacterium Rickettsiella in the Polar Seabird Tick Ixodes uriae. *Microb Ecol,* 71(3), pp. 761-770, doi:10.1007/s00248-015-0702-8.

Mackevicius-Dubickaja, V., Gottlieb, Y., White, J. A. & Doremus, M. R., 2025. Wolbachia feminizes a spider host with assistance from co-infecting symbionts. *Environ Microbiol,* 27(7), pp. e70149, doi:10.1111/1462-2920.70149.

Table S2 Effects of exposure to elevated temperature during ontogeny on symbiont transmission across generations in Mermessus fradeorum. Cool-lines (teal) were continuously reared at 20°C, while Warm-lines (orange) were exposed to an elevated temperature during F1 generation ontogeny. The column on the left indicates symbionts present in parental (P) lines. The diagnostic and dPCR were used to assess the infection status of warm- and cool-treated mothers and a subset of their offspring from each generation. Transmission rates were calculated by dividing the number of infected offspring by the total number of offspring tested per brood.

|  | **P** | | **F1** | | **F2** | | **F3** | |
| --- | --- | --- | --- | --- | --- | --- | --- | --- |
|  | **Cool** | **Hot** | **Cool** | **Hot** | **Cool** | **Hot** | **Cool** | **Hot** |
| *N=* | **15** | **15** | **20** | **13** | **69** | **50** | **12** | **4** |
| **RTW123** | *Rickettiella* | | **100%** | **100%** | **100%** | **100%** | **100%** | **100%** |
|  | *Tisiphia* | | **100%** | **100%** | **100%** | **0%** | **100%** |  |
|  | *Wolbachia 1* | | **100%** | **100%** | **100%** | **76%** | **100%** | **100%** |
|  | *Wolbachia 2* | | **100%** | **100%** | **100%** | **86%** | **100%** | **100%** |
|  | *Wolbachia 3* | | **100%** | **100%** | **90%** | **82%** | **92%** | **100%** |
| *N=* |  | **1** |  | **1** |  | **8** |  |  |
| **RW123** | *Rickettsiella* | |  | **100%** |  | **100%** |  |  |
|  | *Wolbachia1* | |  | **100%** |  | **100%** |  |  |
|  | *Wolbachia2* | |  | **100%** |  | **88%** |  |  |
|  | *Wolbachia3* | |  | **100%** |  | **100%** |  |  |
| *N=* | **5** | **2** | **7** | **1** | **6** | **3** | **12** |  |
| **RTW12** | *Rickettsiella* | | **100%** | **100%** | **100%** | **100%** | **100%** |  |
|  | *Tisiphia* | | **100%** | **100%** | **100%** | **0%** | **100%** |  |
|  | *Wolbachia 1* | | **100%** | **100%** | **100%** | **100%** | **100%** |  |
|  | *Wolbachia 2* | | **100%** | **100%** | **100%** | **100%** | **100%** |  |
| *N=* |  | **5** |  | **6** |  | **6** |  | **16** |
| **RTW1** | *Rickettsiella* | |  | **100%** |  | **100%** |  | **100%** |
|  | *Tisiphia* | |  | **100%** |  | **0%** |  |  |
|  | *Wolbachia 1* | |  | **100%** |  | **83%** |  | **88%** |
